# Association between maternal cholesterol level during pregnancy and placental weight and birthweight ratio: data from the Japan Environment and Children’s Study

**DOI:** 10.1186/s12884-023-05810-3

**Published:** 2023-06-30

**Authors:** Naomi Mitsuda, Masamitsu Eitoku, Keiko Yamasaki, Naw Awn J-P, Mikiya Fujieda, Nagamasa Maeda, Narufumi Suganuma, on behalf of the Japan Environment and Children’s Study (JECS) Group, Michihiro Kamijima, Shin Yamazaki, Yukihiro Ohya, Reiko Kishi, Nobuo Yaegashi, Koichi Hashimoto, Chisato Mori, Shuichi Ito, Zentaro Yamagata, Hidekuni Inadera, Takeo Nakayama, Tomotaka Sobue, Masayuki Shima, Hiroshige Nakamura, Koichi Kusuhara, Takahiko Katoh

**Affiliations:** 1 Department of Environmental Medicine, Kochi Medical School, Kochi University, Kochi, Japan; 2 Department of Pediatrics, Kochi Medical School, Kochi University, Kochi, Japan; 3 Department of Obstetrics and Gynecology, Kochi Medical School, Kochi University, Kochi, Japan

**Keywords:** Hypercholesterolemia, Pregnancy, Japan Environment and Children’s Study, JECS, Birthweight, Placental weight, Placental efficiency

## Abstract

**Background:**

Placental weight to birthweight ratio (PW/BW ratio), or its inverse, is used as an indicator of placental efficiency. Past studies have shown an association between an abnormal PW/BW ratio and adverse intrauterine environment, however, no previous studies have examined the effect of abnormal lipid levels during pregnancy on PW/BW ratio. We aimed to evaluate the association between maternal cholesterol levels during pregnancy and placental weight to birthweight ratio (PW/BW ratio).

**Methods:**

This study was a secondary analysis using the data from the Japan Environment and Children’s Study (JECS). 81 781 singletons and their mothers were included in the analysis. Maternal serum total cholesterol (TC), low-density lipoprotein cholesterol (LDL-C), and high-density lipoprotein cholesterol (HDL-C) levels during pregnancy were obtained from participants. Associations between maternal lipid levels and placental weight and PW/BW ratio were assessed by regression analysis using restricted cubic splines.

**Results:**

Dose–response relationships were observed between maternal lipid level during pregnancy and placental weight and PW/BW ratio. High TC and LDL-C levels were associated with heavy placental weight and high PW/BW ratio, i.e., inappropriately heavy placenta for birthweight. Low HDL-C level was also associated with inappropriately heavy placenta. Low TC and LDL-C levels were associated with low placental weight and low PW/BW ratio, i.e., inappropriately light placenta for birthweight. High HDL-C was not associated with PW/BW ratio. These findings were independent of pre-pregnancy body mass index and gestational weight gain.

**Conclusions:**

Abnormal lipid levels such as elevated TC and LDL-C, and low HDL-C level, during pregnancy were associated with inappropriately heavy placental weight.

**Supplementary Information:**

The online version contains supplementary material available at 10.1186/s12884-023-05810-3.

## Background

Placental weight to birthweight ratio (PW/BW ratio), or its inverse, is thought to reflect placental function and condition of the intrauterine environment better than placental weight alone or birthweight alone, and PW/BW ratio is used as an indicator of placental efficiency [1, 2]. An abnormal PW/BW ratio is associated with some “stressful fetal-placental physiologic states” such as maternal smoking and obesity [1]. Past studies have also shown an association between abnormal PW/BW ratio and many adverse birth outcomes such as low Apgar score and fetal death [3–5].

Cholesterol is essential for fetal growth. During pregnancy, maternal cholesterol levels progressively increase as the fetal demand for cholesterol increases [6]. However, abnormally elevated lipid levels of mothers during pregnancy are associated with pregnancy complications and with adverse birth outcomes such as large for gestational age [7–10]. Since placental weight is strongly correlated with birthweight [11], it is presumed that not only birthweight but also placental weight is affected by maternal lipid levels during pregnancy, and it is presumed that elevated lipid levels are associated with heavy placental weight. Furthermore, if abnormal maternal lipid levels are one of the “stressful fetal-placental physiologic states”, maternal lipid levels may also affect PW/BW ratio. However, no previous studies have examined the association between maternal lipid levels and placental weight or placental efficiency. We hypothesize that maternal abnormal lipid levels during pregnancy are associated with abnormal PW/BW ratio. To test this hypothesis, we evaluated the association between maternal plasma lipid levels during pregnancy and PW/BW ratio by using data from a large birth cohort study, the Japan Environment and Children’s Study (JECS).

## Methods

### Study design

The data used in the present study were obtained from the JECS. The JECS, which is a national project funded directly by the Ministry of Environment, is an ongoing birth cohort study being undertaken to elucidate the influence of environmental factors during the fetal period and early childhood on children’s health, with follow-up until age 13. Pregnant women were recruited for JECS between January 2011 and March 2014. The protocol and baseline data of the JECS are described elsewhere [12, 13]. The JECS protocol was reviewed and approved by the Ministry of the Environment's Institutional Review Board on Epidemiological Studies of the Ministry of the Environment, and the Ethics Committees of all participating institutions. The JECS was conducted in accordance with the Declaration of Helsinki and other internationally valid regulations and guidelines, and with written informed consent from all participants.

### Sample selection

For the present study, we used the dataset “jecs-ta-20190930,” which was released in October 2019. Maternal peripheral blood samples were collected at registration in the JECS (early to mid-pregnancy) and during pregnancy (mid- to late pregnancy), and lifestyle and other background information was collected by using self-administered questionnaires distributed to the participating women at recruitment and later in pregnancy. Physicians, midwives/nurses, and/or Research Co-ordinators transcribed Clinical information on the past and present pregnancies and physical status of the participants and their offspring from medical records created at registration and at delivery [12, 13].

The dataset contained 104 062 fetal records, of which 3759 records involved abortion or stillbirth and 1891 records involved multiple births, leaving 98 412 records of singleton live births. From these, we further excluded cases with missing placental weight (*n* = 3996), cases with missing birthweight (*n* = 5), cases outside of ± 4 standard deviations of the mean placental weight in each gestational week (*n* = 112), cases outside of ± 4 standard deviations of the mean birthweight in each gestational week (*n* = 64), cases with missing or undetermined sex (*n* = 5), cases with missing parity (*n* = 2219), births before 30 weeks of gestation or births after 41 weeks of gestation (*n* = 520), cases with missing data on maternal lipid levels during pregnancy (*n* = 9708), and cases with extremely elevated total cholesterol level (≥ 600 mg/dL; indicative of homozygous familial hypercholesterolemia; *n* = 1) [14]. In total, 81 781 subjects were enrolled (Fig. S1).

### Exposure: maternal lipid levels during pregnancy

The main measures used were maternal total cholesterol (TC), low-density lipoprotein cholesterol (LDL-C), and high-density lipoprotein cholesterol (HDL-C) during pregnancy. Non-fasting maternal blood samples were collected by medical staff when the pregnant women visited Co-operating healthcare providers in early to mid-pregnancy. TC, LDL-C, and HDL-C levels were assayed by a commercial clinical laboratory (SRL, Inc., Tokyo, Japan) along with other biomarkers. TC was determined by using an enzymatic method, LDL-C by using an accelerator selective detergent, and HDL-C by using a liquid selective detergent; all three assays were performed on a Hitachi 7700 Series [15]. Since maternal lipid levels during pregnancy are strongly corelated with gestational age at blood sampling, we used gestational age–adjusted lipid levels, which were calculated as the residuals from the regression model with each lipid level as the dependent variable plus predicted lipid levels for the mean gestational age at blood sampling of the study population [16].

### Outcomes: placental weight and PW/BW ratio

The untrimmed placentas were weighted by a midwife shortly after delivery, with the membranes and umbilical cord attached. The placental weight and birthweight were measured in grams by using a medical scale. PW/BW ratio was calculated as placental weight divided by birthweight. Since there are no standardized reference values for placental weight or PW/BW ratio for the Japanese population, we applied Cole’s LMS method to construct gestational age-, sex-, and parity-specific percentile charts of placental weight and PW/BW ratio [17, 18]. The LMS parameters describe the skewness (*L*, lambda), median (*M*, mu), and coefficient of variation (*S*, sigma) for the growth measurements in each age group. The LMS method assumes that after Box-Cox power transformation, the data for each of a series of age groups are normalized, allowing for a standardized percentile curve to be produced from growth measurements with a skewed distribution. We estimated (sample size–weighted) *L*, *M*, and *S* from placental weight and PW/BW ratio data using the *VGAM* package in R [19]. The constructed parity and sex-specific and PW/BW ratio curves and placental weight curves are shown in Figures S2 and S3, respectively. The LMS parameters and percentile values of placental weight and PW/BW ratio in relation to the gestational age are shown in Tables S1–S4. These analyses were performed with R version 4.1.1.

### Potential covariates

Based on the literature and considering the causal relationships between exposure and outcome variables, the following factors were considered as confounders and covariates: maternal age; pre-pregnancy body mass index (BMI); maternal weight gain during pregnancy, maternal smoking habit during pregnancy (1, never; 2, previously did, but quit before realizing current pregnancy; 3, previously did, but quit after realizing current pregnancy; 4, currently smoking); maternal education [1, junior high school or high school; 2, Technical junior college, Technical/vocational college, or Associate degree; 3, Bachelor’s degree or Graduate degree (Master’s/Doctor’s)]; maternal physical activity (PA) before pregnancy; hypertensive disorder of pregnancy; and gestational diabetes mellitus [5, 7, 8, 10]. PA was assessed by using the Japanese short version of the International Physical Activity Questionnaire, calculated as metabolic equivalent of a task (MET-min/week), and categorized into quartiles [20, 21]. Information on these covariates were transcribed from medical records or from the responses to the questionnaires that were distributed to the mothers during pregnancy.

### Statistical analyses

Descriptive statistics included means and standard deviations (SDs) for continuous variables, and numbers and percentages for categorical variables. The associations of TC, LDL-C, and HDL-C with placental weight and PW/BW ratio were assessed by multivariate regression analyses using restricted cubic splines with knots at the 5th, 35th, 65th, and 95th percentiles of the distribution of each lipid levels [22]; these were the recommended knot locations for the percentiles used [23]. Gestational age-, sex-, and parity-specific z-scores of PW/BW ratio or those of placental weight were assessed as outcome variables. First, adjustments were made for maternal age, pre-pregnancy BMI, gestational weight gain, maternal smoking habit, maternal educational level, and maternal PA before pregnancy. Then, we adjusted the regression models for gestational complications (e.g., hypertensive disorder of pregnancy and gestational diabetes mellitus) to assess whether these factors influenced the association between lipid levels and PW/BW ratio. In addition, we conducted analyses stratified by sex because several studies have shown that the placenta responds to various maternal stressors in a sex-specific way [24, 25]. A significance level of 0.05 was used in these analyses. All analyses were performed using Stata 13.1 (Stata Corp, College Station, Texas).

## Results

Table 1 shows the baseline characteristics of the mothers. The mean (SD) gestational age at blood sampling was 16.0 (3.3) weeks. The mean (SD) TC, LDL-C, and HDL-C levels were 199.8 (34.8) mg/dL, 76.8 (13.7) mg/dL, and 108.2 (28.3) mg/dL, respectively. Table 2 shows the neonatal characteristics according to quintile for adjusted maternal lipid level. Birthweight, placental weight, and PW/BW ratio increased with increasing maternal TC level, whereas gestational age decreased.

**Table 1:** Baseline characteristics of participants

**Timing of blood sampling, weeks**	16.0 (3.3)
**Maternal age at delivery, years**	31.3 (5.0)
**Pre-pregnancy BMI, kg/m**^**2**^	21.2 (3.3)
**Weight gain during pregnancy, kg**	10.3 (4.7)
**Parity**	
Primipara	32,076 (39.2%)
**Maternal education**	
Junior high school or high school	29,241 (35.8%)
Technical junior college, Technical/vocational college, or Associate degree	34,176 (41.8%)
Bachelor’s degree or Graduate degree (Master’s/Doctor’s)	16,947 (20.7%)
**Maternal smoking during pregnancy**	
Never	47,029 (57.5%)
Previously did, but quit before realizing current pregnancy	19,249 (23.5%)
Previously did, but quit after realizing current pregnancy	10,466 (12.8%)
Currently smoking	3,826 (4.7%)
**Physical activity before pregnancy (MET-min/week)**	
≤ 28.3	21,036 (25.7%)
28.4–125.1	19,474 (23.8%)
125.7–434.1	19,501 (23.9%)
435 ≥	19,586 (24.0%)
**Hypertensive disorder of pregnancy**	
Yes	2,507 (3.1%)
**Gestational diabetes mellitus**	
Yes	2,406 (2.9%)

Data are presented as mean (standard deviation), or *n* (%)

*BMI* Body mass index, *MET* Metabolic equivalent of a task

**Table 2:** Birth outcomes according to total cholesterol level quintile

	**Quintile of total cholesterol level**					
	**Q1 (low)**	**Q2**	**Q3**	**Q4**	**Q5 (high)**	***P* for trend**
Gestational age, weeks	39.4 (1.4)	39.3 (1.4)	39.3 (1.4)	39.3 (1.4)	39.2 (1.4)	< 0.001
Birthweight, g	3003 (395)	3026 (399)	3033 (394)	3049 (392)	3058 (402)	< 0.001
Placental weight, g	547 (103)	555 (103)	560 (105)	565 (104)	573 (107)	< 0.001
PW/BW ratio	0.183 (0.028)	0.184 (0.028)	0.185 (0.029)	0.186 (0.028)	0.188 (0.029)	< 0.001

Data are presented as mean (standard deviation). PW/BW ratio, ratio of placental weight to birthweight

**Fig. 1:**
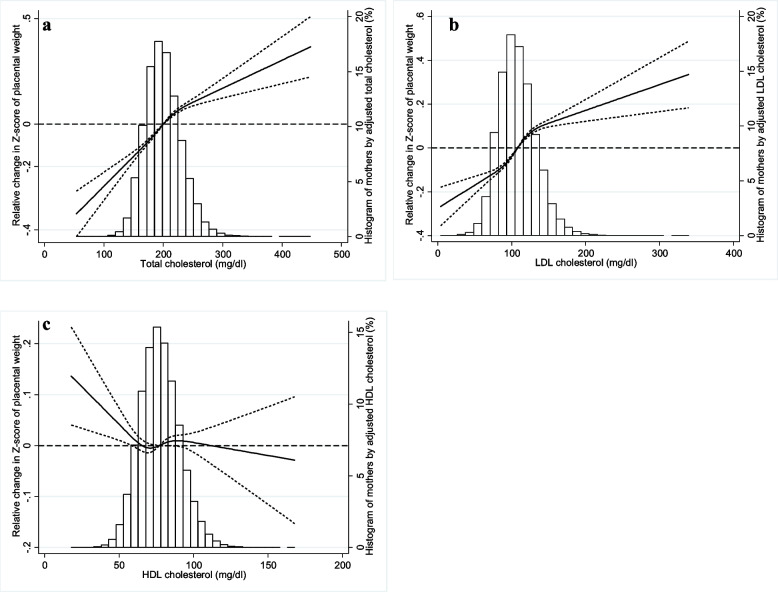
Association between maternal lipid level during pregnancy (**a**: Total cholesterol, **b**: low-density lipoprotein [LDL] cholesterol, and **c**: high-density lipoprotein [HDL] cholesterol) and placental weight (Reference: Total cholesterol = 200 mg/dL, LDL cholesterol = 108 mg/dL, and HDL cholesterol = 77 mg/dL). Adjusted for maternal age, pre-pregnancy body mass index, gestational weight gain, smoking during pregnancy, physical activity before pregnancy, and maternal educational level. Dashed lines indicate 95% confidence interval. Histogram shows distribution of mothers by adjusted lipid level during pregnancy

**Fig. 2:**
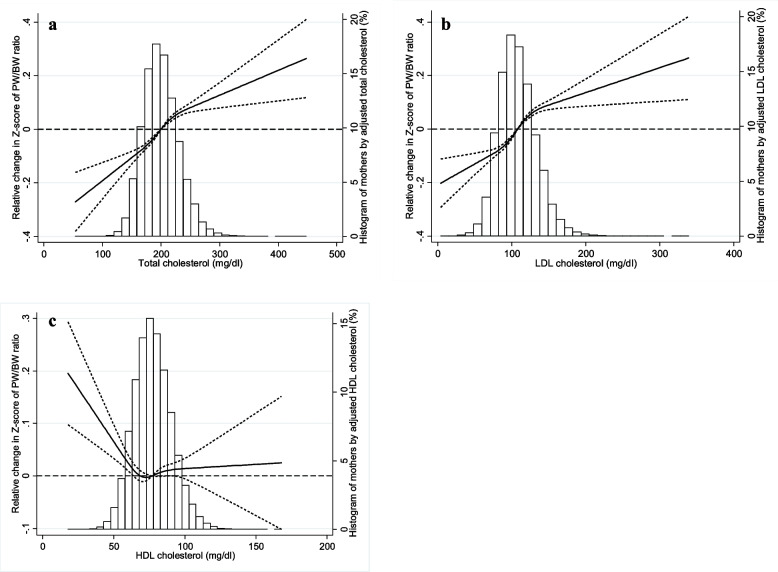
Association between maternal lipid level during pregnancy (**a**: Total cholesterol, **b**: low-density lipoprotein [LDL] cholesterol, and **c**: high-density lipoprotein [HDL] cholesterol) and ratio of placental weight to birthweight (Reference: Total cholesterol = 200 mg/dL, LDL cholesterol = 108 mg/dL, and HDL cholesterol = 77 mg/dL). Adjusted for maternal age, pre-pregnancy body mass index, gestational weight gain, smoking during pregnancy, physical activity before pregnancy, and maternal educational level. Dashed lines indicate 95% confidence interval. Histogram shows distribution of mothers by adjusted lipid level during pregnancy

Figures 1 and 2 show the associations between lipid levels during pregnancy and placental weight and PW/BW ratio, together with the distributions of mothers by lipid level. The z-score of placental weight (Fig. 1a, b) and that of PW/BW ratio (Fig. 2a, b) increased with increased maternal TC and LDL-C. In contrast, the z-score of placental weight and that of PW/BW ratio decreased with increasing HDL-c until close to the mean, after which the z-scores somewhat flattened (Figs. 1c and 2c). Additional adjustments for gestational complications had little effect on the associations between lipid levels and PW/BW ratio (Figure S4). Stratifying by sex showed no marked differences between the sexes (Figure S5).

## Discussion

Our findings demonstrate a dose–response relationship between maternal lipid levels during pregnancy and placental weight and PW/BW ratio. High TC, high LDL-C, and low HDL-C were associated with heavy placental weight and high PW/BW ratio, i.e., inappropriately heavy placenta for birthweight. Low TC and low LDL-C were associated with low placental weight and low PW/BW ratio, i.e., inappropriately light placenta for birthweight.

Placental weight is highly correlated with birthweight [11]. In addition, PW/BW ratio decreases according to gestational age, and the heavier the birthweight, the lower the PW/BW ratio tends to be [26, 27]. However, our findings showed that high TC or LDL-C level was associated with heavy placental weight and increased PW/BW ratio but also decreased gestational age. These findings were independent of pre-pregnancy BMI, gestational weight gain, and gestational complications that tend to coexist with abnormal lipid levels. Past studies have shown that high PW/BW ratio is associated with certain maternal factors such as maternal smoking during pregnancy, maternal anemia, and maternal obesity, which are linked to an adverse intrauterine environment [28–30]. Among these factors, the same combination as seen in the pregnant women with elevated lipid levels in the present study, i.e., heavy birthweight, heavy placental weight, and high PW/BW ratio, has been reported in maternal obesity [5, 31]. Elevated lipid levels are more common in obese or overweight women; however, our results were obtained after adjustment for pre-pregnancy BMI and gestational weight gain. Therefore, our findings suggest that abnormal lipid levels alone alter placental weight and PW/BW ratio independent of maternal obesity or abnormal gestational weight gain. The underlying mechanism by which PW/BW ratio is altered by maternal lipid levels is unknown. However, the placenta is known to alter its function and structure in response to changes of the intrauterine environment. Past studies have shown increased oxidative stress in the placenta and impaired placental microvascular endothelial cell function in mothers with gestational hypercholesterolemia [32, 33]. These changes may be associated with increased placental weight and decreased placental efficiency induced by maternal abnormal lipid levels.

Our findings also showed that hypolipidemia was associated with low PW/BW ratio, i.e., inappropriately low placental weight for birthweight. Low serum cholesterol level is correlated with poor nutritional status. Maternal undernutrition has been shown to impair placental development and function [34]. A previous study has shown that the lifestyle changes associated with Ramadan reduce placental weight but maintain birth weight, which is interpreted as a placental response to the mother’s limited ability to deliver nutrients to the fetus [35]. Similarly, our present finding may also reflect a placental response to a suboptimal nutritional status of mothers with hypolipidemia.

Several studies have shown that the placenta responds to various maternal stressors in a sex-specific way [24, 25]; however, sex had little effect on the association between maternal lipid level and PW/BW ratio in the present study population.

The main strengths of the present study are its large sample size and amount of information, which allowed us to examine not only TC levels but also LDL-C, and HDL-C levels, and allowed us to control for confounding factors such as pre-pregnancy BMI and gestational weight gain. In addition, to the best of our knowledge, this is the first study to assess the impact of maternal lipid levels on placental weight and PW/BW ratio.

The present study has several limitations. First, maternal lipid levels increase during pregnancy and there was a wide range in gestational age at blood sampling in this study population. However, the impact of this variation in gestational age was minimized by using gestational age–adjusted lipid levels. Second, maternal lipid levels were measured only once during pregnancy, which did not allow us to assess the impact of pre-pregnancy lipid levels or change of lipid levels during pregnancy on PW/BW ratio. Third, we could only assess non-fasting blood samples. Because triglycerides are greatly influenced by diet, we could not assess the association between triglycerides and PW/BW ratio. Fourth, the prevalence of gestational complications is lower than those reported in past studies, which suggests that participants of the present study may be biased towards relatively healthy individuals [36, 37]. Finally, the way of measuring placental weight may have differed among Co-operating healthcare providers, although, in Japan, placentas are usually weighed with the membranes and umbilical cord intact. However, even if placental weight is more prone to measurement error than birthweight, measurement error alone is unlikely to explain the observed associations between PW/BW ratio and lipid levels.

## Conclusions

Here, we found that abnormal lipid levels such as elevated TC and LDL-C, and low HDL-C level, during pregnancy were associated with high PW/BW ratio, i.e., inappropriately heavy placental weight. In addition, too low TC and LDL-C during pregnancy were associated with low PW/BW ratio, i.e., inappropriately light placental weight. These findings were independent of maternal obesity, abnormal gestational weight gain, or gestational complications. Although further studies are needed to establish the causal effect of maternal abnormal lipid level on placental function, our study suggest that maternal abnormal lipid levels lead to suboptimality of intrauterine environment and cause morphological changes of the placenta. Our findings also imply the importance of monitoring that maternal lipid levels are in the correct range.

## Supplementary Information

**Additional file 1: Figure S1. **Flow chart for selection of participants from JECS. **Figure S2. **Parity- and sex-specific placental weight/birth weight (PW/BW) curves (a: male, primiparous; b: male, multiparous; c: female, primiparous; d: female, multiparous). **Figure S3. **Parity- and sex-specific placental weight curves (a: male, primiparous; b: male, multiparous; c: female, primiparous; d: female, multiparous). **Figure S4. **Association between maternal lipid level during pregnancy (a: Total cholesterol, b: LDL cholesterol, and c: HDL cholesterol) and PW/BW ratio(Reference: Total cholesterol = 200 mg/dl, LDL cholesterol = 108 mg/dl, and HDL cholesterol = 77 mg/dl). **Figure S5. **Association between maternal lipid level during pregnancy and PW/BW ratio stratified by sex (a: Total cholesterol, male; b: total cholesterol, female; c: LDL cholesterol, male; d: LDL cholesterol, female; e: HDL cholesterol, male; e: HDL cholesterol, female) (Reference: total cholesterol = 200 mg/dl, LDL cholesterol = 108 mg/dl, and HDL cholesterol = 77 mg/dl).

**Additional file 2: Table S1. **Descriptive statistics and LMS parameters for PW/BW ratio (males). **Table S2. **Descriptive statistics and LMS parameters for PW/BW ratio (females). **Table S3. **Descriptive statistics and LMS parameters for placental weight (males). **Table S4. **Descriptive statistics and LMS parameters for placental weight (females).

## Data Availability

Data are unsuitable for public deposition due to ethical restrictions and legal framework of Japan. It is prohibited by the Act on the Protection of Personal Information (Act No. 57 of 30 May 2003, amendment on 9 September 2015) to publicly deposit the data containing personal information. Ethical Guidelines for Medical and Health Research Involving Human Subjects enforced by the Japan Ministry of Education, Culture, Sports, Science and Technology and the Ministry of Health, Labour and Welfare also restricts the open sharing of the epidemiologic data. All inquiries about access to data should be sent to: jecs-en@nies.go.jp. The person responsible for handling enquiries sent to this e-mail address is Dr Shoji F. Nakayama, JECS Programme Office, National Institute for Environmental Studies.

## Abbreviations

BMI: Body mass index
CI: Confidence interval
JECS: Japan Environment and Children’s Study
HDL-C: High-density lipoprotein cholesterol
LDL-C: Low-density lipoprotein cholesterol
PA: Physical activity
PW/BW ratio: Ratio of placental weight to birthweight
TC: Total cholesterol
