# Supplementary material for: Association between maternal cholesterol level during pregnancy and placental weight and birthweight ratio: data from the Japan Environment and Children’s Study
Source: BMC Pregnancy Childbirth. 2023 Jun 30;23:484. doi: 10.1186/s12884-023-05810-3 (PMC10311780; doi:10.1186/s12884-023-05810-3)
Supplement: Supplementary file 1 — Additional file 1: Figure S1. Flow chart for selection of participants from JECS. Figure S2. Parity- and sex-specific placental weight/birth weight (PW/BW) curves (a: male, primiparous; b: male, multiparous; c: female, primiparous; d: female, multiparous). Figure S3. Parity- and sex-specific placental weight curves (a: male, primiparous; b: male, multiparous; c: female, primiparous; d: female, multiparous). Figure S4. Association between maternal lipid level during pregnancy (a: Total cholesterol, b: LDL cholesterol, and c: HDL cholesterol) and PW/BW ratio(Reference: Total cholesterol = 200 mg/dl, LDL cholesterol = 108 mg/dl, and HDL cholesterol = 77 mg/dl). Figure S5. Association between maternal lipid level during pregnancy and PW/BW ratio stratified by sex (a: Total cholesterol, male; b: total cholesterol, female; c: LDL cholesterol, male; d: LDL cholesterol, female; e: HDL cholesterol, male; e: HDL cholesterol, female) (Reference: total cholesterol = 200 mg/dl, LDL cholesterol = 108 mg/dl, and HDL cholesterol = 77 mg/dl). [file 12884_2023_5810_MOESM1_ESM.docx]

Total fetal records in JECS, *n* = 104 062

- Abortion, stillbirth, or missing birth status (*n* = 3759)
- Multiple gestations or missing gestation status (*n* = 1891)

Eligible subjects, singleton live births, *n* = 98 412

- Missing placental weight (*n* =3996)
- Missing birth weight (*n* = 5)
- Outside of ±4 standard deviations from the mean placental weight (*n* = 112) or mean birthweight (*n* = 64) in each gestational week
- Missing or undetermined sex of newborn (*n* = 5)
- Missing parity (*n* = 2219)
- Preterm (<30 weeks), or post-term (>41 weeks) (*n* = 520)
- No data on lipid level or blood sampling after delivery (n = 9708)
- Extremely elevated total cholesterol level (> 600mg/dl) (n = 1)

Participants in this analysis, *n* = 81 781

**Figure S1. Flow chart for selection of participants from JECS**


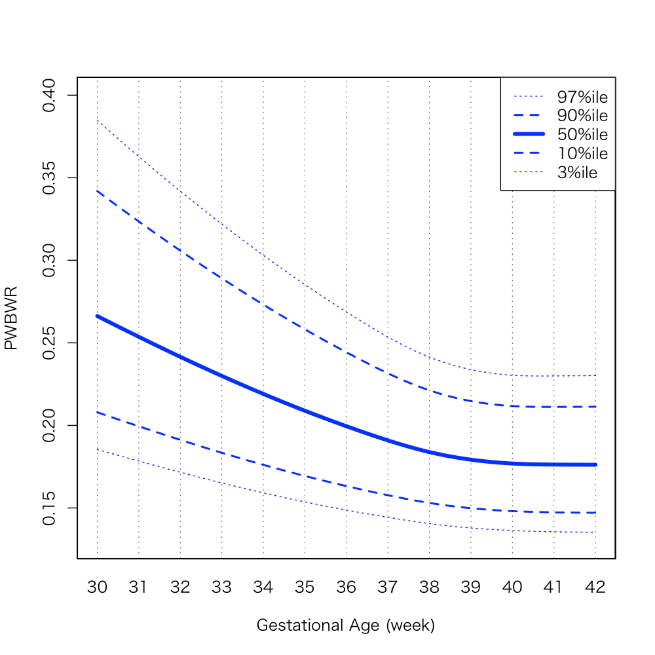

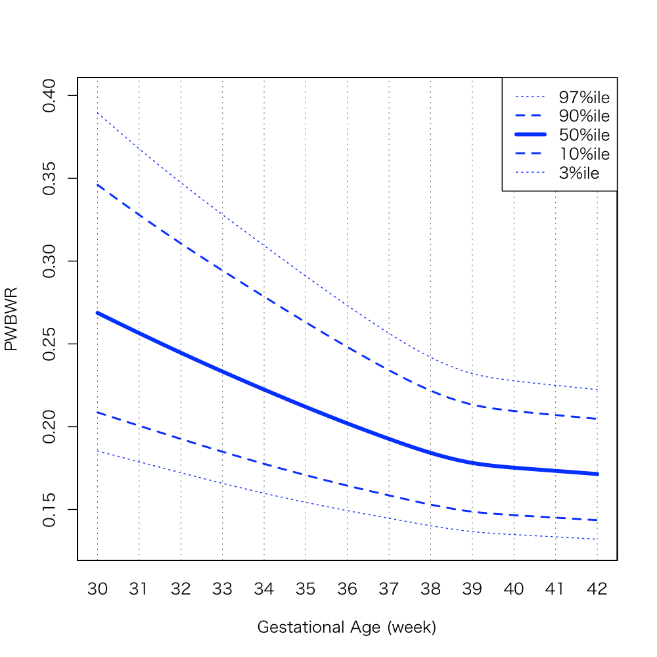


b

a


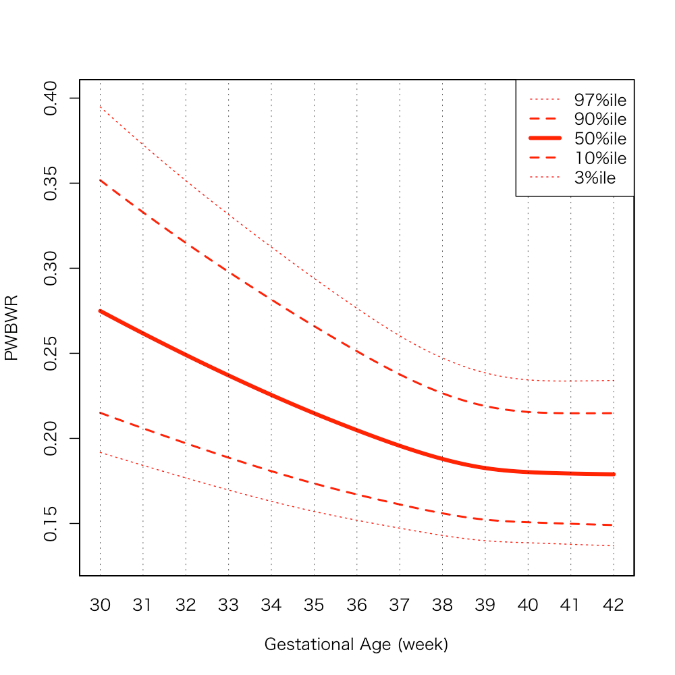

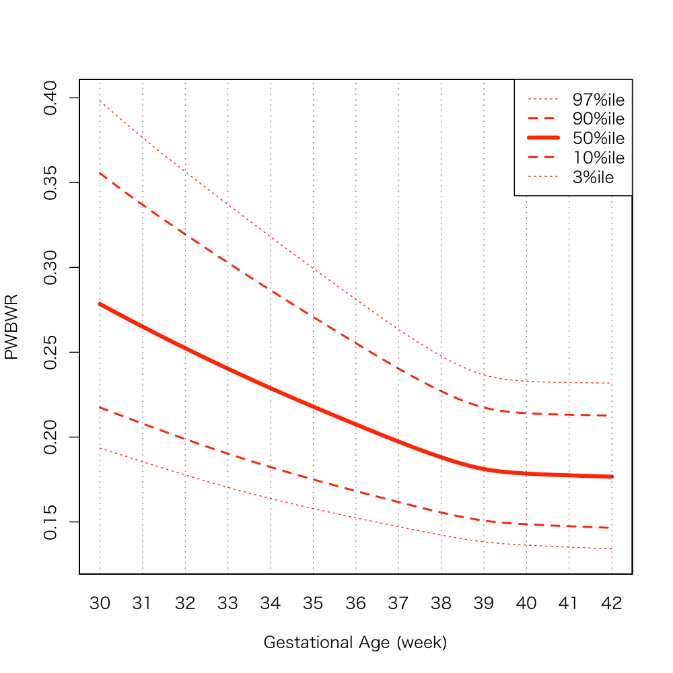


d

c

**Figure S2. Parity- and sex-specific placental weight/birth weight (PW/BW) curves (a: male, primiparous; b: male, multiparous; c: female, primiparous; d: female, multiparous)**


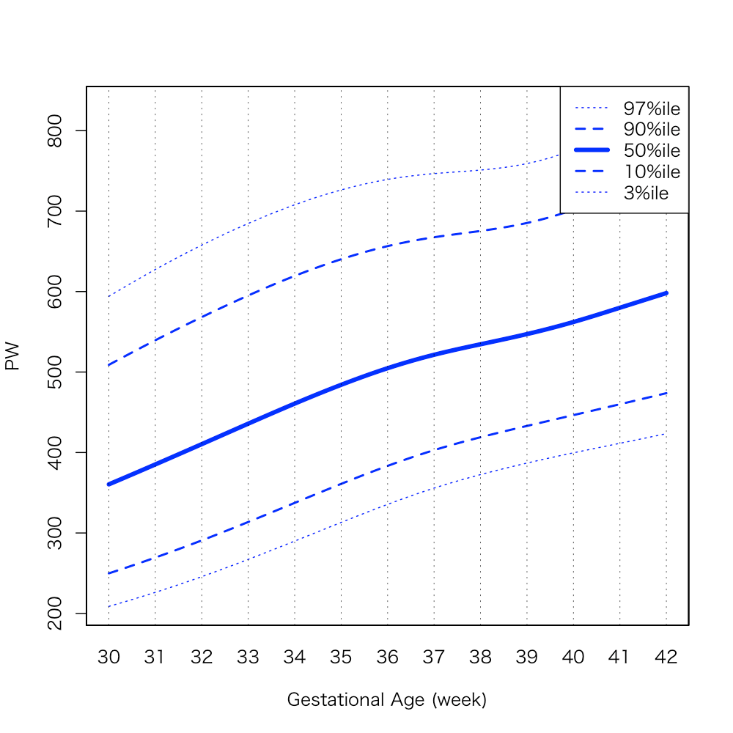

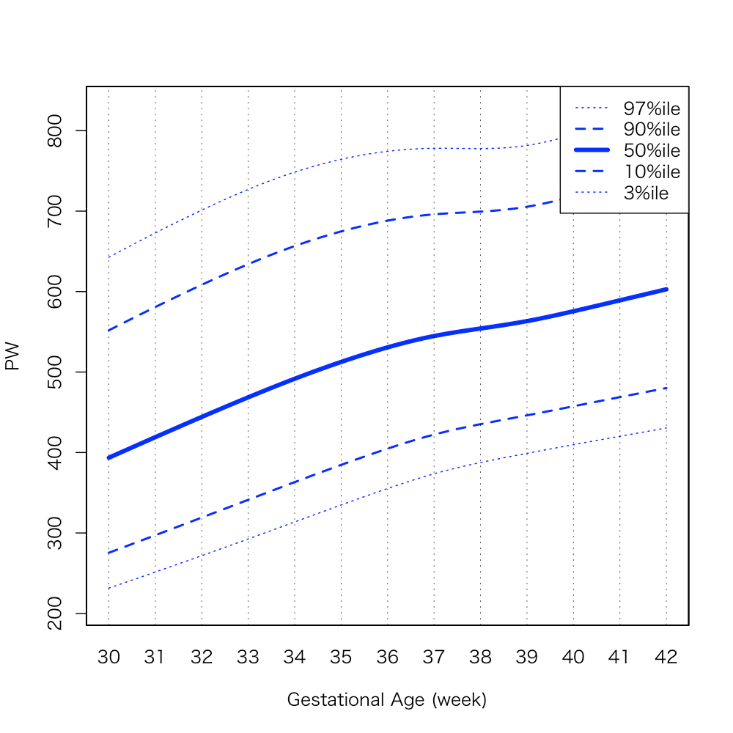


**b**

**a**


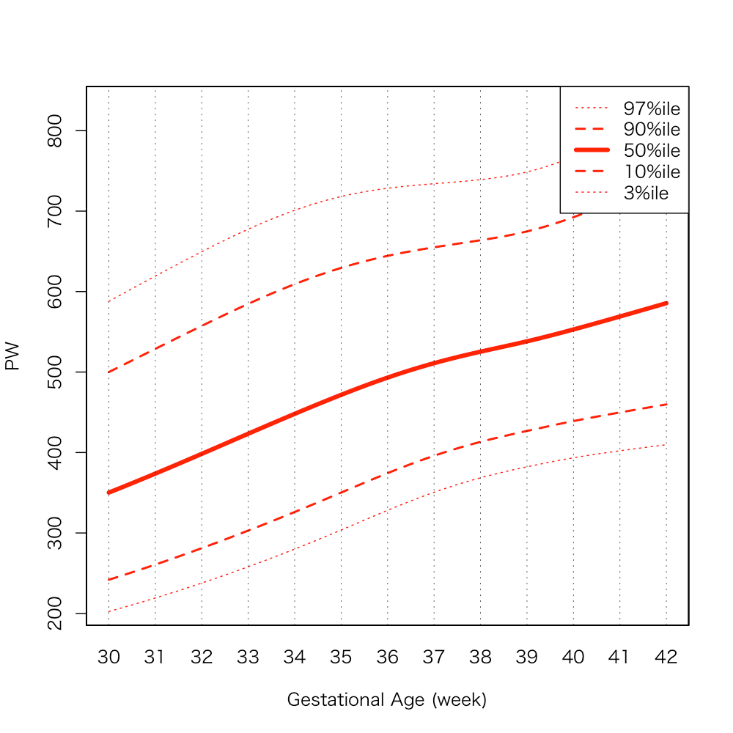

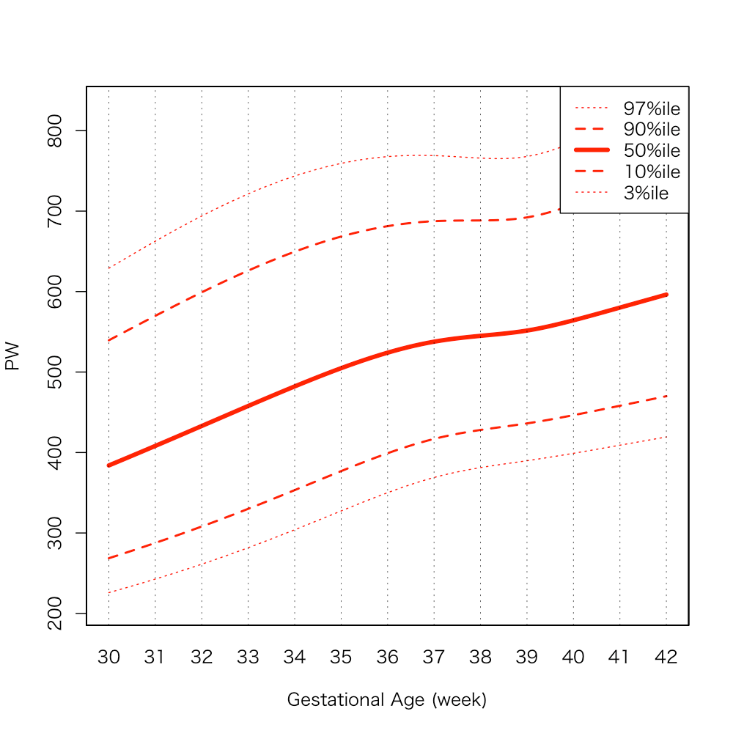


**d**

**c**

**Figure S3. Parity- and sex-specific placental weight curves (a: male, primiparous; b: male, multiparous; c: female, primiparous; d: female, multiparous)**

**a**

**a**

**b**

**c**

**Figure S4. Association between maternal lipid level during pregnancy (a: Total cholesterol, b: LDL cholesterol, and c: HDL cholesterol) and PW/BW ratio** (Reference: Total cholesterol = 200 mg/dl, LDL cholesterol = 108 mg/dl, and HDL cholesterol = 77 mg/dl).

Adjusted for maternal age, pre-pregnancy BMI, gestational weight gain, smoking during pregnancy, physical activity before pregnancy, maternal educational level, gestational hypertension, and gestational diabetes. Dashed lines represent 95% confidence interval. Histogram displays distribution of mothers by adjusted lipid level during pregnancy.

**b**

**a**

**c**

**d**

**f**

**e**

**Figure S5. Association between maternal lipid level during pregnancy and PW/BW ratio stratified by sex (a: Total cholesterol, male; b: total cholesterol, female; c: LDL cholesterol, male; d: LDL cholesterol, female; e: HDL cholesterol, male; e: HDL cholesterol, female)** (Reference: total cholesterol = 200 mg/dl, LDL cholesterol = 108 mg/dl, and HDL cholesterol = 77 mg/dl).

Adjusted for maternal age, pre-pregnancy BMI, gestational weight gain, smoking during pregnancy, physical activity before pregnancy, and maternal educational level. Dashed lines represent 95% confidence interval. Histogram displays distribution of mothers by adjusted lipid level during pregnancy.
