# Supplementary material for: Association between maternal cholesterol level during pregnancy and placental weight and birthweight ratio: data from the Japan Environment and Children’s Study
Source: BMC Pregnancy Childbirth. 2023 Jun 30;23:484. doi: 10.1186/s12884-023-05810-3 (PMC10311780; doi:10.1186/s12884-023-05810-3)
Supplement: Supplementary file 2 — Additional file 2: Table S1. Descriptive statistics and LMS parameters for PW/BW ratio (males). Table S2. Descriptive statistics and LMS parameters for PW/BW ratio (females). Table S3. Descriptive statistics and LMS parameters for placental weight (males). Table S4. Descriptive statistics and LMS parameters for placental weight (females). [file 12884_2023_5810_MOESM2_ESM.docx]

**Table S1. Descriptive statistics and LMS parameters for PW/BW ratio (males)**

| Gestational age (weeks) | Primiparous | | | | | | Multiparous | | | | | |
| --- | --- | --- | --- | --- | --- | --- | --- | --- | --- | --- | --- | --- |
|  | L | M | S | Percentile | | | L | M | S | Percentile | | |
|  |  |  |  | 10th | 50th | 90th |  |  |  | 10th | 50th | 90th |
| 30 | −0.04828 | 0.266226 | 0.194081 | 0.20791 | 0.266226 | 0.34192 | 0.004042 | 0.268726 | 0.197449 | 0.208621 | 0.268726 | 0.346057 |
| 31 | −0.04828 | 0.253655 | 0.188679 | 0.199452 | 0.253655 | 0.3235 | 0.004042 | 0.256472 | 0.191787 | 0.20056 | 0.256472 | 0.327892 |
| 32 | −0.04828 | 0.24152 | 0.183167 | 0.191241 | 0.24152 | 0.305831 | 0.004042 | 0.24464 | 0.186529 | 0.192602 | 0.24464 | 0.310666 |
| 33 | −0.04828 | 0.229967 | 0.177457 | 0.183416 | 0.229967 | 0.289054 | 0.004042 | 0.233285 | 0.181386 | 0.184878 | 0.233285 | 0.294302 |
| 34 | −0.04828 | 0.219059 | 0.171251 | 0.176096 | 0.219059 | 0.273139 | 0.004042 | 0.222402 | 0.175618 | 0.177562 | 0.222402 | 0.278508 |
| 35 | −0.04828 | 0.208888 | 0.164486 | 0.169366 | 0.208888 | 0.258184 | 0.004042 | 0.21193 | 0.16861 | 0.170729 | 0.21193 | 0.263023 |
| 36 | −0.04828 | 0.199502 | 0.157302 | 0.163238 | 0.199502 | 0.244302 | 0.004042 | 0.201975 | 0.160586 | 0.164393 | 0.201975 | 0.248106 |
| 37 | −0.04828 | 0.19092 | 0.149812 | 0.157708 | 0.19092 | 0.231537 | 0.004042 | 0.192625 | 0.152087 | 0.158501 | 0.192625 | 0.234059 |
| 38 | −0.04828 | 0.183841 | 0.143795 | 0.153026 | 0.183841 | 0.221225 | 0.004042 | 0.18418 | 0.144992 | 0.152937 | 0.18418 | 0.221773 |
| 39 | −0.04828 | 0.179238 | 0.140372 | 0.149845 | 0.179238 | 0.214733 | 0.004042 | 0.178112 | 0.140794 | 0.148697 | 0.178112 | 0.213317 |
| 40 | −0.04828 | 0.17691 | 0.139473 | 0.148068 | 0.17691 | 0.211698 | 0.004042 | 0.175243 | 0.139299 | 0.146583 | 0.175243 | 0.20948 |
| 41 | −0.04828 | 0.176269 | 0.140457 | 0.147346 | 0.176269 | 0.211199 | 0.004042 | 0.173313 | 0.138785 | 0.145064 | 0.173313 | 0.207036 |

PW/BW ratio: ratio of placental weight to birthweight.

LMS parameters describe the skewness (L, lambda), the median (M, mu), and the coefficient of variation (S, sigma) for the growth measurements in each gestational age group.

**Table S2. Descriptive statistics and LMS parameters for PW/BW ratio (females)**

| Gestational age (weeks) | Primiparous | | | | | | Multiparous | | | | | |
| --- | --- | --- | --- | --- | --- | --- | --- | --- | --- | --- | --- | --- |
|  | L | M | S | Percentile | | | L | M | S | Percentile | | |
|  |  |  |  | 10th | 50th | 90th |  |  |  | 10th | 50th | 90th |
| 30 | −0.01399965 | 0.2748886 | 0.19203165 | 0.21501127 | 0.2748886 | 0.35173896 | 0.048073 | 0.278445 | 0.191832 | 0.217438 | 0.278445 | 0.355534 |
| 31 | −0.01399965 | 0.26170957 | 0.18738191 | 0.20592222 | 0.26170957 | 0.33287915 | 0.048073 | 0.265091 | 0.188219 | 0.207982 | 0.265091 | 0.336937 |
| 32 | −0.01399965 | 0.24906188 | 0.18274946 | 0.19713359 | 0.24906188 | 0.31491067 | 0.048073 | 0.252392 | 0.185086 | 0.198825 | 0.252392 | 0.319526 |
| 33 | −0.01399965 | 0.23698828 | 0.17813103 | 0.18868719 | 0.23698828 | 0.29787095 | 0.048073 | 0.240317 | 0.1814 | 0.190219 | 0.240317 | 0.302821 |
| 34 | −0.01399965 | 0.22551731 | 0.1729741 | 0.18074097 | 0.22551731 | 0.28158006 | 0.048073 | 0.228845 | 0.176397 | 0.182317 | 0.228845 | 0.286543 |
| 35 | −0.01399965 | 0.21475191 | 0.16673029 | 0.17349156 | 0.21475191 | 0.2659949 | 0.048073 | 0.217948 | 0.170216 | 0.175031 | 0.217948 | 0.270767 |
| 36 | −0.01399965 | 0.20479336 | 0.15921068 | 0.16704373 | 0.20479336 | 0.25122027 | 0.048073 | 0.207468 | 0.162916 | 0.168197 | 0.207468 | 0.255371 |
| 37 | −0.01399965 | 0.19564403 | 0.15144958 | 0.1611716 | 0.19564403 | 0.23761494 | 0.048073 | 0.197346 | 0.154742 | 0.161693 | 0.197346 | 0.240406 |
| 38 | −0.01399965 | 0.18790099 | 0.14561009 | 0.15595253 | 0.18790099 | 0.22650481 | 0.048073 | 0.188094 | 0.147535 | 0.155556 | 0.188094 | 0.227048 |
| 39 | −0.01399965 | 0.18254296 | 0.14186857 | 0.15223185 | 0.18254296 | 0.21899069 | 0.048073 | 0.181187 | 0.142873 | 0.150749 | 0.181187 | 0.217419 |
| 40 | −0.01399965 | 0.18018401 | 0.13959135 | 0.15070266 | 0.18018401 | 0.21552922 | 0.048073 | 0.178499 | 0.1424 | 0.148604 | 0.178499 | 0.214065 |
| 41 | −0.01399965 | 0.17933728 | 0.14054165 | 0.14981237 | 0.17933728 | 0.21477847 | 0.048073 | 0.177431 | 0.14381 | 0.147446 | 0.177431 | 0.213166 |

PW/BW ratio: ratio of placental weight to birthweight.

LMS parameters describe the skewness (L, lambda), the median (M, mu), and the coefficient of variation (S, sigma) for the growth measurements in each gestational age group.

**Table S3. Descriptive statistics and LMS parameters for placental weight (males)**

| Gestational age (weeks) | Primiparous | | | | | | Multiparous | | | | | |
| --- | --- | --- | --- | --- | --- | --- | --- | --- | --- | --- | --- | --- |
|  | L | M | S | Percentile | | | L | M | S | Percentile | | |
|  |  |  |  | 10th | 50th | 90th |  |  |  | 10th | 50th | 90th |
| 30 | 0.170457 | 360.4468 | 0.277288 | 249.8268 | 360.4468 | 508.9531 | 0.154404 | 393.5256 | 0.270896 | 275.4282 | 393.5256 | 551.879 |
| 31 | 0.170457 | 385.1866 | 0.270554 | 269.4357 | 385.1866 | 539.4756 | 0.154404 | 419.0756 | 0.261169 | 297.1957 | 419.0756 | 580.7914 |
| 32 | 0.170457 | 410.4019 | 0.261157 | 290.7676 | 410.4019 | 568.286 | 0.154404 | 444.2184 | 0.2515 | 319.1654 | 444.2184 | 608.4188 |
| 33 | 0.170457 | 435.8426 | 0.249616 | 313.6673 | 435.8426 | 595.1181 | 0.154404 | 468.6016 | 0.241578 | 341.2153 | 468.6016 | 634.0804 |
| 34 | 0.170457 | 460.7683 | 0.236846 | 337.387 | 460.7683 | 619.4517 | 0.154404 | 491.66 | 0.23084 | 363.2112 | 491.66 | 656.5921 |
| 35 | 0.170457 | 484.1216 | 0.22332 | 361.0165 | 484.1216 | 640.1931 | 0.154404 | 512.5553 | 0.219165 | 384.6235 | 512.5553 | 674.7621 |
| 36 | 0.170457 | 504.7687 | 0.209724 | 383.3608 | 504.7687 | 656.4834 | 0.154404 | 530.6835 | 0.20687 | 404.832 | 530.6835 | 688.1438 |
| 37 | 0.170457 | 521.4469 | 0.19681 | 402.9464 | 521.4469 | 667.5123 | 0.154404 | 544.751 | 0.194866 | 422.2746 | 544.751 | 696.0107 |
| 38 | 0.170457 | 534.5208 | 0.186152 | 418.9788 | 534.5208 | 675.3368 | 0.154404 | 554.1248 | 0.184964 | 435.2418 | 554.1248 | 699.3816 |
| 39 | 0.170457 | 547.1885 | 0.178986 | 433.0309 | 547.1885 | 685.2627 | 0.154404 | 563.2962 | 0.178639 | 446.1817 | 563.2962 | 705.4157 |
| 40 | 0.170457 | 562.2016 | 0.176332 | 446.489 | 562.2016 | 701.7624 | 0.154404 | 575.551 | 0.176032 | 457.4697 | 575.551 | 718.4399 |
| 41 | 0.170457 | 579.9206 | 0.177247 | 459.9994 | 579.9206 | 724.6982 | 0.154404 | 589.1277 | 0.175094 | 468.8448 | 589.1277 | 734.5331 |

LMS parameters describe the skewness (L, lambda), the median (M, mu), and the coefficient of variation (S, sigma) for the growth measurements in each gestational age group

**Table S4. Descriptive statistics and LMS parameters for placental weight (females)**

| Gestational age (weeks) | Primiparous | | | | | | Multiparous | | | | | |
| --- | --- | --- | --- | --- | --- | --- | --- | --- | --- | --- | --- | --- |
|  | L | M | S | Percentile | | | L | M | S | Percentile | | |
|  |  |  |  | 10th | 50th | 90th |  |  |  | 10th | 50th | 90th |
| 30 | 0.106926 | 350.2368 | 0.2831 | 241.9128 | 350.2368 | 499.9741 | 0.154404 | 393.5256 | 0.270896 | 275.4282 | 393.5256 | 551.879 |
| 31 | 0.106926 | 373.7948 | 0.27581 | 260.7049 | 373.7948 | 528.8242 | 0.154404 | 419.0756 | 0.261169 | 297.1957 | 419.0756 | 580.7914 |
| 32 | 0.106926 | 398.339 | 0.266905 | 281.1362 | 398.339 | 557.3809 | 0.154404 | 444.2184 | 0.2515 | 319.1654 | 444.2184 | 608.4188 |
| 33 | 0.106926 | 423.3476 | 0.256425 | 302.9777 | 423.3476 | 584.7436 | 0.154404 | 468.6016 | 0.241578 | 341.2153 | 468.6016 | 634.0804 |
| 34 | 0.106926 | 448.0512 | 0.243786 | 326.0811 | 448.0512 | 609.2485 | 0.154404 | 491.66 | 0.23084 | 363.2112 | 491.66 | 656.5921 |
| 35 | 0.106926 | 471.6823 | 0.22862 | 350.2441 | 471.6823 | 629.4195 | 0.154404 | 512.5553 | 0.219165 | 384.6235 | 512.5553 | 674.7621 |
| 36 | 0.106926 | 493.102 | 0.211829 | 374.364 | 493.102 | 644.4003 | 0.154404 | 530.6835 | 0.20687 | 404.832 | 530.6835 | 688.1438 |
| 37 | 0.106926 | 511.0061 | 0.196345 | 395.958 | 511.0061 | 655.031 | 0.154404 | 544.751 | 0.194866 | 422.2746 | 544.751 | 696.0107 |
| 38 | 0.106926 | 525.3168 | 0.18491 | 413.2182 | 525.3168 | 663.8264 | 0.154404 | 554.1248 | 0.184964 | 435.2418 | 554.1248 | 699.3816 |
| 39 | 0.106926 | 538.1657 | 0.178666 | 426.8136 | 538.1657 | 674.7741 | 0.154404 | 563.2962 | 0.178639 | 446.1817 | 563.2962 | 705.4157 |
| 40 | 0.106926 | 552.9127 | 0.177689 | 439.0724 | 552.9127 | 692.4178 | 0.154404 | 575.551 | 0.176032 | 457.4697 | 575.551 | 718.4399 |
| 41 | 0.106926 | 569.0264 | 0.181199 | 449.7893 | 569.0264 | 715.7327 | 0.154404 | 589.1277 | 0.175094 | 468.8448 | 589.1277 | 734.5331 |

LMS parameters describe the skewness (L, lambda), the median (M, mu), and the coefficient of variation (S, sigma) for the growth measurements in each gestational age group.
